# The Altitude of Coffee Cultivation Causes Shifts in the Microbial Community Assembly and Biochemical Compounds in Natural Induced Anaerobic Fermentations

**DOI:** 10.3389/fmicb.2021.671395

**Published:** 2021-05-20

**Authors:** Silvia Juliana Martinez, João Batista Pavesi Simão, Victor Satler Pylro, Rosane Freitas Schwan

**Affiliations:** ^1^Department of Biology, Federal University of Lavras (UFLA), Lavras, Brazil; ^2^Technology and Coffee Growing Program, Federal Institute of Espírito Santo, Alegre, Brazil

**Keywords:** target sequencing, Caparaó region, microbial community, altitude, coffee fermentation

## Abstract

Coffee harvested in the Caparaó region (Minas Gerais, Brazil) is associated with high-quality coffee beans resulting in high-quality beverages. We characterize, microbiologically and chemically, fermented coffees from different altitudes through target NGS, chromatography, and conventional chemical assays. The genera *Gluconobacter* and *Weissella* were dominant in coffee’s fruits from altitudes 800 and 1,000 m. Among the Eukaryotic community, yeasts were the most dominant in all altitudes. The most dominant fungal genus was *Cystofilobasidium*, which inhabits cold environments and resists low temperatures. The content of acetic acid was higher at altitudes 1,200 and 1,400 m. Lactic acid and the genus *Leuconostoc* (Pearson: 0.93) were positively correlated. The relative concentration of volatile alcohols, especially of 2-heptanol, was high at all altitudes. Bacteria population was higher in coffees from 800 m, while at 1,000 m, fungi richness was favored. The altitude is an important variable that caused shifts in the microbial community and biochemical compounds content, even in coffees belonging to the same variety and cultivated in the same region under SIAF (self-induced anaerobic fermentation) conditions. Coffee from lower altitudes has higher volatile alcohols content, while high altitudes have esters, aldehydes, and total phenolics contents.

## Introduction

The Caparaó is a region located in a mountainous territory shared by two Brazilian states, Minas Gerais and Espírito Santo (Assis et al., 2017; Campanha et al., 2017), and known for producing high-quality coffees. The coffee plants are owned by family farms grown at different altitudes and microclimates (Campanha et al., 2017). Ninety percent of the Caparaó region’s production is of *Coffea arabica*, representing 40% of total production in the Espírito Santo state (Santos et al., 2017), and 75% of them are processed by the natural method (Paschoa et al., 2017).

Crop growing environment, plants genetic traits, and post-harvesting processes are among the essential drivers of coffee quality (De Bruyn et al., 2017; Borém et al., 2019), meaning that coffee is a *terroir* product, and care is needed to obtain specialty coffee beverages. Three methods are commonly used to process coffee: natural (dry), wet, and pulped natural (semi-dry). The natural method is the oldest process that uses whole intact fruits, directly placed on cement patios or suspended platforms for fermentation and drying until reaching 11–12% moisture (Schwan et al., 2012). In the wet method, fruits are depulped, then fermented in tanks with water, and placed directly for drying. While the pulped natural is a mixture of both methods where fruits are depulped and placed directly for fermentation and drying. Each method has shown differences in sensory perception and microbiota dynamics (Silva et al., 2000, 2008a; Avallone et al., 2001; Evangelista et al., 2014a,b; Bressani et al., 2018). A more recent method known as self-induced anaerobic fermentation (SIAF) showed promising results in Da Mota et al. (2020) and Martins et al. (2020).

During fermentation, microorganisms consume carbohydrates or other organic compounds and proliferate (Silva et al., 2000; Silva, 2015). Most microorganisms that participate in the process come from the environment like soil, air, plants, and other sources (Silva et al., 2008a; Silva, 2015). Yeasts from the genera *Saccharomyces*, *Pichia*, *Candida*, *Kluyveromyces*, *Hanseniaspora*, and bacteria belonging to *Leuconostoc*, *Lactobacillus*, *Bacillus*, *Flavobacterium*, *Serratia*, *Pseudomonas*, and *Weissella* are often found while fermentation in the different post-harvest processes (Silva et al., 2000; Avallone et al., 2001; Masoud et al., 2004; Vilela et al., 2010; Silva, 2015).

Microbial communities usually change in response to the environmental conditions where fermentations are carried out and affect coffee quality. Those conditions include temperature, moisture, and altitude (Borém et al., 2019; Martins et al., 2020). Bertrand et al. (2006) observed that green coffee beans from the variety Caturra grew at high altitudes and processed via the wet method in Costa Rica have high caffeine and fats and low trigonelline contents. A study with Ethiopian arabica green coffee beans showed that an increase in altitude decrease caffeine and chlorogenic acids contents, while sucrose, acidity, and flavor increase (Worku et al., 2018). In a Brazil, research conducted in the Matas de Minas region showed that yellow and red Catuaí coffee varieties from higher altitudes produce higher quality coffee beans (Silveira et al., 2016).

Further research regarding altitude vs. compounds content variation is needed because they directly influence the beverage flavor. For example, organic acids mainly affect the sweet flavor (Galli and Barbas, 2004) and acidity (Ribeiro et al., 2017). Bioactive compounds trigonelline and chlorogenic acids are precursors of volatile compounds that contribute to roasted coffees taste and aroma (Ribeiro et al., 2016), and volatile alcohol precursors produce rose-like and fruity-like flavors (Lee et al., 2015).

Recent advances in Next-Generation Sequencing (NGS) are now allowing a deep microbiota characterization during the fermentative process under different conditions in several countries (Cao et al., 2017; de Oliveira Junqueira et al., 2019; Zhang et al., 2019; Elhalis et al., 2020; Pothakos et al., 2020), but few studies have been carried out with Brazilian coffees.

The present study aimed to characterize the dominant microbial communities of bacteria and fungi present in self-induced anaerobic fermentations containing different altitudes coffees performed in the Caparaó region through a metataxonomic approach. Moreover, this study aimed to evaluate the effect of altitude and microbiota profile on the biochemical compounds profile (organic acids, bioactives, and volatiles) during the fermentative process.

## Materials and Methods

### Pilot Study On-Farm: Coffee Process and Fermentation

Ripe fruits of *Coffea arabica* cv Catuaí Vermelho IAC 44 were manually collected from different altitudes: 800, 1,000, 1,200, and 1,400 m, at the Caparaó region, located in Minas Gerais and Espírito Santo, Brazil. The coffee fruits were processed using the natural method. Then the fruits were transferred into 20 L bioreactors (polypropylene food buckets with lids), with following the bioreactors were closed for SIAF. Fermentations were performed in triplicate.

The fermentative processes for all coffees from different altitudes were carried out simultaneously in close batches at a farm located at 1,200 m to avoid any environmental interference and favor controlled conditions. The bioreactors were placed under an open storage house built with fences for fermentation and suspended terraces. Before filling the bioreactors with coffee, portable data loggers (INKBIRD) were placed inside the bioreactors to register the mass temperature during fermentation. Fermentation lasted 72 h, and sub-samples of approximately 100 g were taken after 48 h of fermentation for dominant microbiota profiling and metabolites evaluation. Fruits’ initial sugar content (Brix degree-°Bx) was measured with a refractometer (Sigma-Aldrich, St. Louis, MO, United States).

### Composition and Abundance of Bacteria and Fungi Communities

#### DNA Extraction

Total DNA was extracted from 48 h fermented coffee fruits collected in fields at 800, 1,000, 1,200, 1,400 m of altitude. One hundred grams of coffee fruits were vortexed in 50 ml of sterilized Milli-Q water for 10 min to detach the fruits’ microbial cells. Then the resulted suspension was transferred to another tube and centrifuged (12,745 RCF for 10 min at 4°C) to separate the supernatant and obtain a pellet. After the supernatant was discarded, 30 mg of the remaining pellet was used for DNA extraction with the QIAamp DNA Mini Kit, following the “DNA Purification from Tissues” protocol (Qiagen, Hilden, Germany). The purity of the extracted DNA was checked with a Nanodrop Lite spectrophotometer (Nanodrop Technologies, Wilmington, DE, United States) (260/280 nm ratio), and it was quantified by Qubit^®^ 4.0 fluorometer using the dsDNA HS Assay kit (Invitrogen^TM^) according to the manual. The DNA integrity was also confirmed by electrophoresis in a 0.8% agarose gel with 1 X TAE buffer.

#### Illumina High-Throughput Sequencing of Bacterial/Archaeal 16S rRNA Genes and Fungal Internal Transcribed Spacer (ITS)

The NGS Soluções Genômicas performed sample preparation for sequence and sequencing in Piracicaba-São Paulo, Brazil. The V3-V4 regions of the 16S rRNA gene of bacteria and the ITS1 and ITS2 regions of fungi were amplified from the total DNA extracted. We used the primers 341F (5′-CCTACGGGNGG CWGCAG-3′) and 806R (5′-GACTACHVGGGTATCTAATCC-3′) (Klindworth et al., 2013) for bacteria/archaea, and the ITS1f (5′-CTTGGTCATTTAGAGGAAGTAA-3′) and ITS2 (5′-GCTGCGTTCTTCATCGATGC-3′) (Gardes and Bruns, 1993; Smith and Peay, 2014) for fungi. Samples were paired-ended sequenced (2 × 250 bp) on an Illumina MiSeq platform using the V2 kit (Illumina Inc.).

#### Data Analysis

The raw.fastq files were used to build a table of amplicon sequence variants (ASVs) with dada2 version 1.12 (Callahan et al., 2016). Briefly, using default parameters, the raw data quality was evaluated, filtered, and trimmed. The filtering parameters [maxN = 0, truncQ = 2, rm.phix = TRUE, maxEE = (2,2), and truncLen (235, 230)] were applied before inputting the filtered reads into dada2’s parametric error model. The truncLen parameter was not applied for ITS1 and ITS2 reads since the expected sequence length is variable for fungi. Later, the forward and reverse reads were merged to obtain a full denoised sequence, and a higher-resolution table of amplicon sequence variants (ASVs) was constructed. Only ASVs with total abundances higher than 0.1% are reported. Chimeric sequences were detected and removed. Taxonomy was assigned to each ASV using the RDP ribosomal RNA gene database (version 11.5) for the 16S rRNA gene and with UNITE database (version 8.2) for fungal ITS. Sequences were matched the reference sequence with 100% identity.

### Biochemical Analysis

#### Organic Acids Evaluation

Organic acids of coffee fruits were evaluated after 48 h of fermentation. Three grams of coffee fruits were vortexed in Falcon tubes containing 20 mL of 16 mM perchloric acid and Milli-Q water at room temperature (25°C) for 10 min. The resulted suspension (without the fruits) was transferred to another tube, centrifuged at 12,745 RCF for 10 min at 4°C to obtain the supernatant. The supernatant was transferred to a new tube, and then its pH value was adjusted to 2.11 using perchloric acid and recentrifuged under the same conditions. The supernatant from the second centrifugation was filtered through a 0.22 μm cellulose acetate membrane (Merck Millipore, Germany) and directly injected (20 μL) chromatographic column.

The samples were analyzed using a high-performance liquid chromatography (HPLC) system (Shimadzu Corp., Japan) equipped with a detection system consisting of an UV–Vis detector (SPD 10Ai) and a Shimpack SCR-101H (7.9 mm 30 cm) column operating at 50°C, which was used to achieve chromatographic separation of water-soluble acids at a flow rate of 0.6 mL min^–1^. The acids were identified by comparison with the retention times of authentic standards. The quantification was performed using calibration curves constructed with standard compounds [malic and citric acid were purchased from Merck (Darmstadt, Germany), lactic and tartaric acid were purchased from Sigma-Aldrich (Saint Louis, MO, United States), acetic and succinic acids were purchased from Sigma-Aldrich, isobutyric and butyric acid were purchased from Riedel-deHaen (Seelze, Germany)]. All analyses were performed in duplicate.

#### Caffeine, Trigonelline, and Chlorogenic Acids by HPLC

The identification of caffeine, chlorogenic acid [5-CGA], and trigonelline was made using a Shimadzu liquid chromatography system (Shimadzu Corp., Japan) equipped with a C18 column, following the protocol proposed by Malta and Chagas (2009). 0.5 g of grounded coffee fruits were placed in tubes containing 50 mL Milli-Q water and boiled for 3 min to extract total compounds. Then the suspension was filtered through a 0.22 μm cellulose acetate membrane (Merck Millipore). Identification and quantitative analysis were performed using caffeine calibration curves, trigonelline, and 5-CGA (Sigma-Aldrich). All analyses were performed in duplicate.

#### Total Polyphenols and Antioxidant Activity

Coffee samples were defatted following the methodology described by Batista et al. (2016). One hundred fruits were grounded with liquid nitrogen per sample, then 4 g were weighted. Following, 20 mL of n-hexane (Merck) was added into the 4 g, vortexed for 5 min, and centrifuged at 4,200 × g for 10 min/4°C to separate the lipids from the grounded sample left in the supernatant. After discarding the supernatant, the same procedure was repeated three times. The resulted lipid-free samples were air-dried for 24 h to evaporate the residual organic solvent.

The polyphenols and antioxidants were extracted, according to Kim et al. (2018), with minor modifications. Fifty milliliter of distilled water at 90°C were added in a tube containing 2.75 g lipid-free ground coffee. Then, the mixture was left standing at room temperature (25°C) for 20 min. After that period, the mixture was filtered through a Whatman No. 2 filter paper.

##### Determination of Total Polyphenol Content (TPC)

The total polyphenol content (TPC) was determined by a spectrophotometric assay (UV-VIS Spectrum SP-2000 UV, Biosystems) following the Follin—Ciocalteau methodology (Singleton and Rossi, 1965). In brief, 500 μL of coffee extract, 2.5 mL of Folin–Ciocalteau reagent (10%), and 2.0 mL of Na_2_CO_3_ (4% w/v) were homogenized and incubated at room temperature (25°C), in the dark for 120 min. The absorbance of the samples was measured at 750 nm. The TPC concentrations were calculated based on the standard curve of gallic acid (ranging from 10 to 100 μg mL^–1^) and expressed as milligrams of gallic acid equivalents per gram of ground coffee (mg GAE g^–1^). All analyses were performed in triplicate.

##### Antioxidant Activity Assays

Two different methodologies were applied to measure the antioxidant activity of coffee extracts. In the first one, the 2,2-diphenyl-1-picryl-hydrazyl-hydrate (DPPH) radical scavenging assay was performed as follows: 0.1 mL of coffee extract was added to 3.9 mL of the DPPH radical solution (0.06 mM) and incubated at room temperature (25°C), in the dark for 120 min, then the absorbance was measured at 515 nm (Spectrophotometer UV-Vis Spectrum^®^ SP-2000UV, Shanghai, China). Trolox was used as a standard. A calibration curve (*y* = −0.0004*x* + 0.6636) was assembled using a range of 10, 20, 30, 40, 50 and 60 μM Trolox with linearity *R*^2^ = 0.9999 (Batista et al., 2016). The results were expressed as μM Trolox Equivalents (TE) per gram of ground coffee (μM TE g^–1^).

The second assay was performed with a 2,2′-azinobis (3-ethylbenzothiazoline-6-suslfonic acid) (ABTS) stock solution reaction (7 mM) with potassium persulfate (140 mM). The mixture was left in the dark at room temperature (25°C) for 16 h before use. The ABTS solution was diluted in ethanol to an absorbance of 0.70 ± 0.05 at 734 nm. Thirty microliters of the coffee extracts were added to 3.0 mL of the ABTS radical solution, and after 6 min, the absorbance was measured. Trolox was used as a standard. A calibration curve (*y* = −0.0003*x* + 0.6802) was assembled using a range of 100, 500, 1,000, 1,500 and 2,000 μM Trolox with linearity of *R*^2^ = 0.9983. The results were expressed as μM Trolox Equivalents (TE) per gram of ground coffee (μM TE g^–1^).

#### Volatile Compounds

Volatile compounds were extracted from 48 h fermented coffee fruits using a headspace solid-phase microextraction (HS-SPME). Coffee fruits (2 g) were macerated with liquid nitrogen and placed in a 15 ml hermetically sealed vial. After equilibration at 60°C for 15 min, the volatile compounds were extracted at 60°C for 30 min. The desorption time on the column was 2 min.

The compounds were analyzed using a Shimadzu QP2010 GC model equipped with mass spectrometry (MS) and a silica capillary Carbo-Wax 20M (30 m × 0.25 mm × 0.25 mm) column. The operation conditions of analysis consisted of maintaining the oven temperature at 50°C for 5 min, then raised to 200°C at 8°C min^–1^ and maintained for 15 min. The injector and detector were kept at 230 and 200°C, respectively, and He carrier gas was maintained at a flow rate of 1.9 ml min^–1^. The volatile compounds were identified by comparing their mass spectra against those available in the NIST11 library. The retention Index (RI) for each compound was calculated using an alkane series (C10–C40) compared with those found in the literature.

### Statistical Analysis

Alpha and beta diversity analyzes were performed for the evaluated microbial communities. Each altitude richness and abundance were used to calculate the bacterial and fungal Shannon and Simpson diversity indices. Moreover, the relative abundance was calculated, and ASVs profiles were clustered for each altitude using the XLSTAT software (Addinsoft, version 2020.1.3). Bray-Curtis-based non-metric multidimensional scaling (NMDS) was used to evaluate the dissimilarities between the fungal community and organic acids and volatile compounds with the XLSTAT software (Addinsoft, version 2020.1.3).

The raw data normal distribution for statistical analysis was evaluated with the Shapiro-Wilk and Anderson-Darling tests. All values in the figures are expressed as averages. Standard deviations were calculated using the XLSTAT software (Addinsoft, version 2020.1.3). The Tukey test was run with *p* ≤ 0.05 in the SISVAR software (Ferreira, 2014) to evaluate the difference in acid concentration, volatiles relative concentration, and antioxidants’ concentration and activity. The Pearson correlation coefficient was used to calculate the correlations between the bacterial genera, acids, and volatile compounds, using Origin software (version 2020). The principal component analysis was run on all altitudes, acids, antioxidants, and volatile compounds using XLSTAT software (Addinsoft, version 2020.1.3).

## Results

### Fruits Initial Sugar Content and Fermentation Temperature

The initial °Brix value from coffee fruits was between 18 and 19.3 (Table 1). The coffee mass temperature varied from 18 to 25°C at 48 h (Table 1). The environmental temperature varied from 8 to 23.1°C and relative moisture varied from 56.1 to 85% during fermentation.

**TABLE 1:** Fruits initial brix, coffee mass temperature, and microbial diversity indices.

**Coffee altitude (m)**	**Initial brix (Bx)**	**Coffee mass temperature (°C)**			**Bacterial diversity indices**		**Eukaryotic diversity indices**	
		**0 h**	**24 h**	**48 h**	**Shannon**	**Simpson**	**Shannon**	**Simpson**
800	18.6 ± 0.6	18 ± 0	22 ± 0.06	23 ± 0.15	2.281 ± 0.2	7.973 ± 1.7	3.334 ± 0.1	14.766 ± 2.0
1,000	18.6 ± 1.5	18 ± 0	24 ± 0.12	25 ± 0.06	2.018 ± 0.5	5.574 ± 2.2	2.721 ± 0.1	5.666 ± 0.3
1,200	19.3 ± 0.6	18 ± 0	20 ± 0.10	21.6 ± 0.06	2.005 ± 0.4	5.004 ± 2.0	0.924 ± 0.3	1.417 ± 0.2
1,400	18 ± 3.0	18 ± 0	22 ± 0.12	23 ± 0.15	1.661 ± 0.5	4.330 ± 1.3	2.983 ± 0.2	9.372 ± 1.0

*Data are expressed as Mean ± SD.*

*SD, Standard deviation.*

### Microbial Community Profile

A total of 63.966, 16.346, 42.238, and 19.727 filtered 16S rRNA partial gene sequences and 104.719, 194.033, 263.884, and 119.571 filtered ITS sequences were obtained for the altitudes 800, 1,000, 1,200, and 1,400 m, respectively.

Among the altitudes, 800 m had the highest bacterial richness with 18 genera assigned, and 1,000 m had the highest fungal richness with 166 species assigned. Table 1 shows the bacterial and fungal diversity indices for all evaluated altitudes. In summary, we observed a tendency to decrease the alpha-bacterial diversity indices with the altitude increase (Table 1).

**FIGURE 1:**
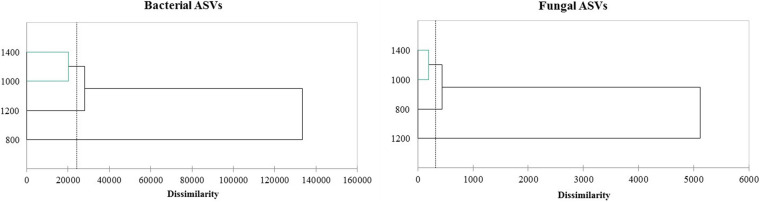
Clustering of ASVs profile from amplicon sequence of the 16S and ITS region.

The altitudes ASVs profiles were clustered, as illustrated in Figure 1, and three groups were obtained for bacteria and fungi. The 800 m bacterial profile was very distant and different from the other altitudes. The 1,400 and 1,000 m profiles were grouped for bacteria and fungi, meaning they were the most similar. On the other hand, the fungal cluster showed that the 1,200 m profile was different from the other altitudes and close to the 800 m profile.

**FIGURE 2:**
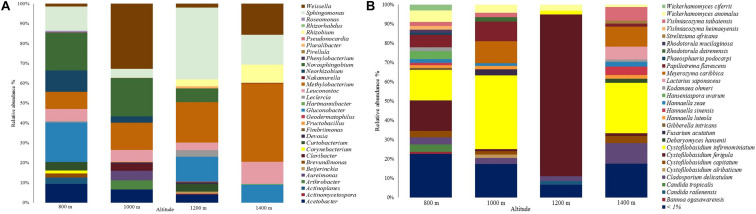
Relative abundance of the microbial communities at different altitudes. **(A)** Bacterial community. **(B)** Fungal community.

A total of 31 genera were assigned in the bacterial community, as shown in Figure 2A. Most sequences in the 800 m sample were assigned to genera *Gluconobacter* (19.8%), *Novosphingobium* (18.9%), and *Sphingomonas* (12.2%). As for the other altitudes, genera *Weissella* (32.7%; 1,000 m), *Sphingomonas* (36.2%; 1,200 m), and *Methylobacterium* (39.4%; 1,400 m) had the highest abundances.

The genera *Actinoplanes*, *Brevundimonas*, *Corynebacterium*, *Roseomonas*, *Phenylobacterium, Pseudonocardia*, and *Rhizorhabdus*, were only identified at 800 m, *Arthrobacter*, *Clavibacter*, *Fructobacillus*, and *Pirellula* were only identified at 1,000 m, *Beijerinckia*, *Pluralibacter*, *Actinomycetospora*, *Geodermatophilus*, and *Fimbriimonas* were only identified at 1,200 m, and *Nakamurella* and *Hartmannibacte*r at 1,400 m. *Sphingomonas*, *Methylobacterium*, *Leuconotoc*, and *Weissella* were found in all altitudes. Genus *Gluconobacter* was only identified in samples at 800, 1,200, and 1,400 m with relative abundances of 19.8, 12.5, and 9%, respectively.

Regarding the fungal community, a total of 223 species were assigned, showing a yeast predominance (Figure 2B). The most abundant species were *Cystofilobasidium infirmominiatum* (15.831%), *Cystofilobasidium ferigula* (15.700%), and *Papiliotrema flavescens* (6.571%) at 800 m. Following, *Cystofilobasidium infirmominiatum* (38.218%), *Meyerozyma caribbica* (11.445%), and *Papiliotrema flavescens* (10.271%) at 1,000 m, *Cystofilobasidium ferigula* (83.857%), *Wickerhamomyces anomalus* (3.216%), and *Cladosporium delicatulum* (2.539%) at 1,200 m, and *Cystofilobasidium infirmominiatum* (26.187%), *Cladosporium delicatulum* (10.817%), and *Meyerozyma caribbica* (10.216%) at 1,400 m. The species that were below 1% relative abundance are available in Supplementary Material 1.

Each altitude had a broad range of distinctive fungal species, which included *Candida sake*, *Sampaiozyma vanillica*, *Apiotrichum laibachii*, and *Citeromyces matritensis* for 800 m, *Fellomyces borneensis*, *Rhodotorula babjevae*, *Rhodotorula taiwanensis*, *Papiliotrema laurentii*, *Candida blattae*, *Wickerhamomyces pijperi*, *Cryptococcus saitoi*, *Rhynchogastrema complexa*, and *Cutaneotrichosporon terricola* for 1,000 m, *Papiliotrema perniciosus*, *Wickerhamomyces sydowiorum*, *Nakazawaea holstii*, and *Eupenidiella venezuelensis* for 1,200 m, and *Neoascochyta paspali*, *Euteratosphaeria verrucosiafricana*, *Sporobolomyces johnsonii*, and *Rhodosporidiobolus ruineniae* for 1,400 m. The rest of the distinctive species identified are available in Supplementary Material 2.

We also identified frequently described species grouped in the species below 1% of abundance (Supplementary Material 1). Those include *Saccharomyces cerevisiae*, which was only identified in altitudes 1,000 (0.006%) and 1,200 m (0.003%), *Candida parapsilosis* in the same altitudes with 0.022 and 0.015%, and *Torulaspora delbrueckii* in altitudes 800, 1,000, and 1,200 m with 0.062, 0.036, and 0.005%. There were other yeasts like *Meyerozyma guilliermondii* (identified at 800 and 1,000 m: 0.904 and 0.130%), *Candida tropicalis* (lower abundances at 1,000, 1,200, and 1,400 m: 0.182, 0.162, and 0.156%), *Debaryomyces hansenii* (lower abundances at 800, 1,000, and 1,200 m: 0.282, 0.398, and 0.869%), *Pichia kluyveri*, *Debaryomyces nepalensis*, *Rhodotorula mucilaginosa* (only in altitudes 1,000, 1,200, and 1,400), *Candida orthopsilosis*, *Candida quercitrusa*, *Fellomyces mexicanus*, *Derxomyces anomalus*, and *Wickerhamomyces lynferdii*.

Filamentous fungi species such as *Aspergillus westerdijkiae*, *Alternaria argyroxiphii*, *Botrytis caroliniana*, *Cladosporium aphidis*, *Cladosporium halotolerans*, *Colletotrichum annellatum*, *Colletotrichum theobromicola*, *Fusarium asiaticum*, *Fusarium delphinoides*, *Fusarium proliferatum*, *Gibberella intricans*, *Lecanicillium antillanum*, *Penicillium kongii*, and *Penicillium solitum* were also identified.

### Organic Acids

#### Effect of Altitude on Acids Content

**FIGURE 3:**
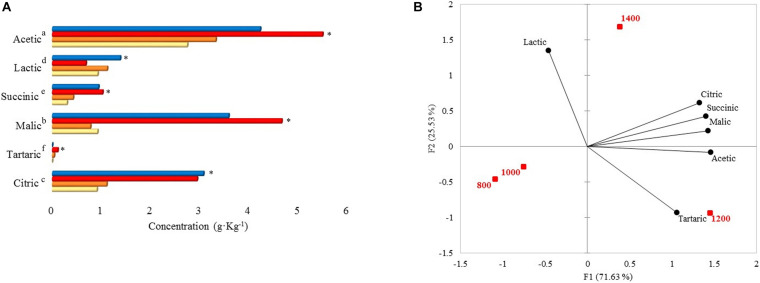
**(A)** Organic acid concentrations at 48 h of fermentation on different altitudes. Each bar represents an altitude: 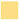 800, 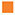 1,000, 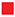 1,200, and 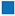 1,400 m. Significant values (*p* ≤ 0.05) are represented in letters, from the highest amount to the lowest. ^∗^Altitude that was statistically significant (*p* ≤ 0.05) and higher in contrast to the other altitudes. Citric, tartaric, malic, succinic, lactic, and acetic standard deviations: 800 m (0.19, not detected, 0.09, 0.01, 0.43, 0.44), 1,000 m (0.46, 0, 0.37, 0.10, 0.73, 1.57), 1,200 m (1.52, 0, 0.87, 0.41, 0.17, 1.95), 1,400 m (2.55, not detected, 1.97, 0.65, 1.11, 1.65). **(B)** Principal component analysis (PCA) plot of organic acids and altitudes.

Acetic, malic, citric, lactic, succinic, and tartaric acid concentrations were statistically different among the altitudes (Figure 3A). Acetic, malic, and citric acid concentrations at 1,400 and 1,200 m were higher than 1,000 and 800 m. When concentrations from 1,400 and 1,200 m were compared with the other altitudes in each acid, there were differences of 0.90–2.75 (acetic), 2.66–3.88 (malic), and 1.84–2.16 (citric) g. Kg^–1^. As for altitudes 1,000 and 800 m, acetic (g. Kg^–1^: 3.32 and 2.74), lactic (g. Kg^–1^: 1.12 and 0.92), citric (g. Kg^–1^: 1.10 and 0.91), and malic acid (g. Kg^–1^: 0.78 and 0.92) were found in higher concentrations than succinic and tartaric acid. Within the acetic acid results, 1,200 m altitude presented the highest content (5.49 g. Kg^–1^), and altitude 800 m presented the lowest content with 2.74 g. Kg^–1^. Malic and succinic acid were significantly higher at 1,200 m (g. Kg^–1^: 4.66 and 1.02). Citric and lactic acid were higher at 1,400 m (3.07 g. Kg^–1^ and 1.38 g. Kg^–1^, respectively). Tartaric acid was only detected at 1,000 and 1,200 m with concentrations of 0.04 and 0.11 g. Kg^–1^, respectively.

The PCA showed that 1,200 m and 1,400 m altitudes were correlated with citric, succinic, malic, and acetic acid (Figure 3B). 1,400 m altitude was characterized by lactic acid, while 1,200 m altitude was characterized by tartaric acid (Figure 3B).

#### Acids Correlation With Bacterial Community and Dissimilarity With Fungal Community

**FIGURE 4:**
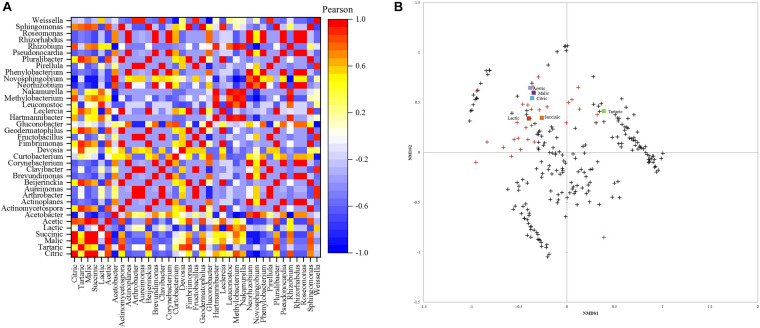
**(A)** Pearson correlation matrix of bacterial genera and acids. **(B)** Non-metric multidimensional scaling (NMDS) using Bray-Curtis dissimilarity for eukaryotic species and organic acids.**+** species below 1% and **
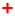
** abundant species above 1%.

The Pearson correlation between acid content and bacterial community is depicted in Figure 4A. *Leuconostoc* showed a high positive correlation (0.93) with lactic acid content. Malic acid had the highest positive correlation (0.87) with the *Sphingomonas* genus. Acetic acid was positively correlated (0.87) with *Sphingomonas* and negatively correlated with *Acetobacter* (−0.64). The genera *Pluralibacter*, *Geodermatophilus*, *Fimbriimonas*, *Beijerinckia*, and *Actinomycetospora*, were highly positively correlated with tartaric acid (0.93 for all). Additionally, succinic and citric acid were highly positively correlated with *Rizhobium* (0.78, 0.87) and *Methylobacterium* with citric acid (0.83).

The NMDS plot (Figure 4B) showed that similar acetic, malic, citric, lactic, and succinic acid contents are shared between the fungal community, mainly in greater abundance. Most species with an abundance below 1% were close and different from the high abundance species and did not affect the acids in concentrations.

### Volatile Compounds

#### Effect of Altitude on Volatiles

A total of 67 volatile compounds were detected. These compounds were classified as alcohols (19), esters (13), aldehydes (10), ketones (6), furans (5), phenols (4), pyrans (3), acids (3), alkanes (2), lactones (1), and pyrazines (1).

**FIGURE 5:**
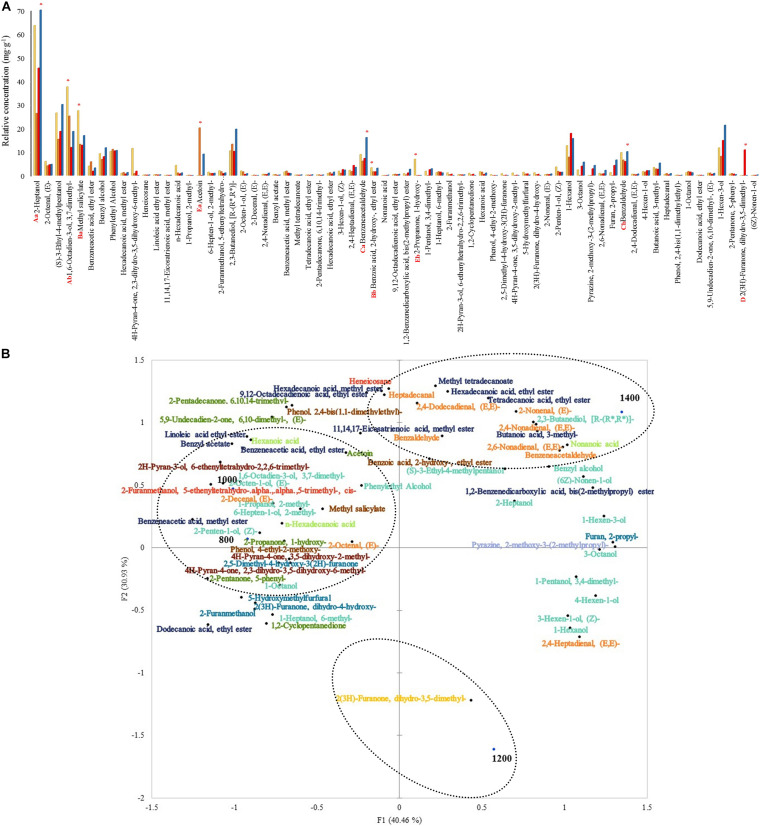
**(A)** Volatile compounds relative concentrations at 48 h of fermentation on different altitudes. Each bar represents an altitude: 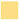 800, 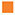 1,000, 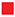 1,200, and 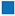 1,400 m. Significant values (*p* ≤ 0.05) are represented in letters. From the highest amount to lowest, different uppercase letters represent the most predominant groups (A: alcohols, B: phenols, C: aldehydes, D: lactones, and E: ketones), and different lowercase letters represent the two most predominant compounds within the groups. * Altitude that was statistically significant (*p* ≤ 0.05) and higher in contrast to the other altitudes. **(B)** Principal component analysis (PCA) plot of volatile compounds and altitudes. Volatile groups: 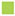 acids,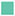 alcohols, 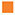 aldehydes, 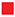 alkanes, 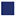 esters, 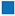 furans, 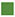 ketones, 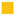 lactones, 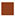 phenols, 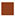 pyrans, 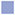 pyrazines.

The total relative concentration of each chemical group was statistically significant. The following chemical groups had the most abundant relative concentrations: alcohols, phenols, aldehydes, lactones, and ketones. The alcohols 2-heptanol and 1,6-octadien-3-ol, 3,7-dimethyl- were the most significant among the other compounds with relative concentrations varying from 70.6 to 26.3 mg. g^–1^ and 37.5 to 11.8 mg. g^–1^ (Figure 5A). The relative concentration of these alcohols was higher at 1,400 and 800 m compared to the other altitudes, respectively. Moreover, methyl salicylate and benzoic acid, 2- hydroxy-, ethyl ester were the most abundant within the phenols group and both in altitudes at 800 m (27.4 and 3.3 mg. g^–1^). Aside from the previous groups, other compounds such as benzeneacetaldehyde, benzaldehyde, 2(3H)-furanone, dihydro-3,5- dimethyl-, acetoin, and 2-propanone, 1-hydroxy- were the most abundant within the aldehydes, lactones, and ketones groups, at altitudes 1,400 (relative concentration: 16.1 and 10.1 mg. g^–1^), 1,200 (10.8 mg. g^–1^), 1,000 (20.1 mg. g^–1^), and 800 (6.9 mg. g^–1^) m. Some compounds were detected only in certain altitudes: 2-propanone, 1-hydroxy- and phenol, 4-ethyl-2-methoxy- at 800 m, 1-propanol, 2-methyl- and 2-decenal, (E)- at 1,000 m, 2(3H)-furanone, dihydro-3,5-dimethyl- at 1,200 m, and non-anoic acid at 1,400 m. Other compounds like (S)-3-ethyl-4-methylpentanol, benzyl alcohol, phenylethyl alcohol, 2,3-butanediol, [R-(R^∗^, R^∗^) ]-, and benzyl acetate were detected.

The PCA graph in Figure 5B showed that around 36% (7) of those alcohols characterized 1,000 and 800 m altitudes from the total volatile alcohols. A total of 1,200 m altitude was characterized by the only lactone 2(3H)-furanone, dihydro-3,5-dimethyl-. A total of 1,400 m altitude was primarily characterized by esters (70%-7 from the total).

#### Volatiles Correlation With Bacterial Community and Dissimilarity With Fungal Community

**FIGURE 6:**
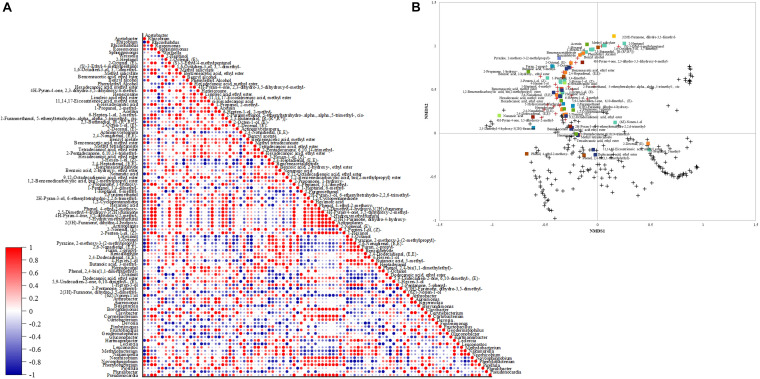
**(A)** Down-stream Pearson correlation matrix of bacterial genera and volatile compounds. **(B)** Non-metric scaling (NMDS) using Bray-Curtis dissimilarity for eukaryotic species and volatiles. Volatile groups: 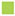 acids,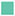 alcohols, 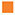 aldehydes, 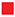 alkanes, 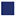 esters, 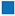 furans, 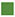 ketones, 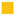 lactones, 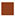 phenols, 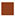 pyrans, 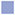 pyrazines. **+** species below 1% and 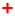 abundant species above 1%.

The Pearson correlation between the volatile compounds and bacterial profile is depicted in Figure 6A. Methyl salicylate and 2-propanone, 1-hydroxy- were positively correlated (1) with the genera only found at 800 m altitudes (*Actinoplanes*, *Brevundimonas*, *Corynebacterium*, *Roseomonas*, *Phenylobacterium*, *Pseudonocardia*, and *Rhizorhabdus*). 2(3H)-furanone, dihydro-3,5-dimethyl- was positively correlated (1) with the genera only found at 1,200 m altitude (*Pluralibacter*, *Geodermatophilus*, *Fimbriimonas*, *Beijerinckia*, and *Actinomycetospora*). The genus *Weisella* had a strong positive correlation (0.99) with the ketone acetoin, 11,14,17-eicosatrienoic acid, methyl ester (0.98), and phenylethyl alcohol (0.97). The highest correlation (0.96) for benzeneacetaldehyde was with the species only found at 1,400 m altitude (*Nakamurella* and *Hartmannibacter*).

The NMDS plot (Figure 6B) showed that the species with the highest abundance within the fungal community might be producing similar contents of detected volatiles. Though not all the high abundance species affect the same volatile groups, some affect alcohols contents instead of aldehydes contents as observed in the plot. Most fungal species below 1% abundance are close and different from high abundance species and are grouped with low content volatiles (from 0 to 0.4 mg. g^–1^) phenol, 2,4-bis(1,1-dimethylethyl)-, phenol, 4-ethyl-2- methoxy-, 11,14,17-eicosatrienoic acid, methyl ester, dodecanoic acid, ethyl ester, 2-decenal, (E)-, 1-propanol, 2- methyl-, and (6Z)-nonen-1-ol.

### Effect of Altitude on Caffeine, Chlorogenic Acids, Trigonelline, Total Phenolics Concentration, and Antioxidant Activity

**FIGURE 7:**
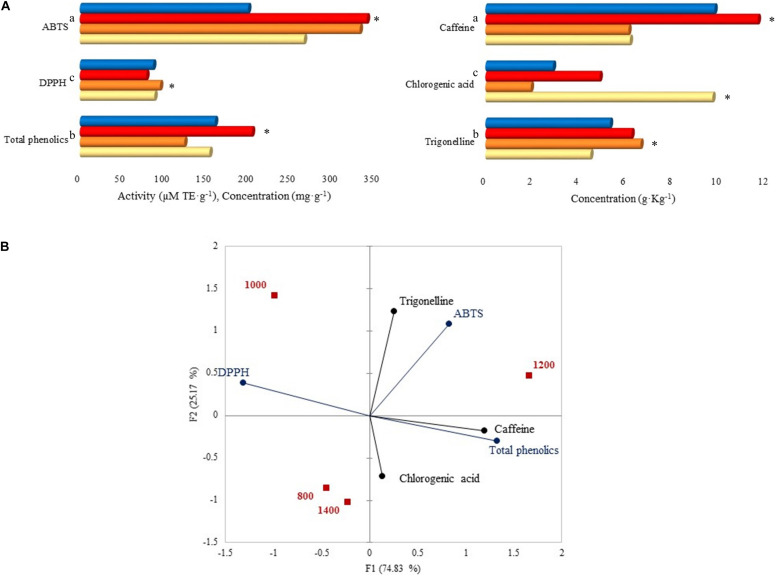
**(A)** Caffeine, chlorogenic acid, trigonelline, total phenolics concentrations, and antioxidant activity by ABTS and DPPH assays. Each bar represents an altitude: 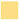 800, 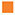 1,000, 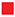 1,200, and 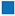 1,400 m. Statistically significant values (*p* ≤ 0.05) are represented in letters, from the highest amount to the lowest. ^∗^Altitude that was statistically significant (*p* ≤ 0.05) and higher in contrast to the other altitudes. Trigonelline, chlorogenic acid, caffeine, total phenolics, DPPH, and ABTS standard deviations: 800 m (1.62, 8.33, 5.21, 0.21, 0, 0.03), 1,000 m (0.78, 0.06, 1.17, 3.72, 0, 21.84), 1,200 m (0.03, 0.01, 0, 3.15, 0, 17.10), 1,400 m (0.08, 0.01, 0.02, 6.60, 0, 0.15). **(B)** Principal component analysis (PCA) plot of antioxidants, antioxidant activity, and altitudes.

Caffeine total concentration was higher (44.5%) than those of trigonelline (29.9%) and chlorogenic acid (25.6%), mainly at 1,200 m with a significant value of 11.64 g. Kg^–1^ (Figure 7A). Among the altitudes, trigonelline concentration was higher at 1,000 m (6.63 g. Kg^–1^), and chlorogenic acid was higher at 800 m (9.70 g. Kg^–1^). Total phenolics concentration was higher at 1,200 m with 203.90 mg. g^–1^, followed by 1,400 m (160.12 mg. g^–1^). Regarding the antioxidant activity, after the ABTS assay, samples at 1,000 and 1,200 m had the highest activity (331.72 and 340.16 μM TE. g^–1^) compared to other altitudes, and after the DPPH assay, the highest value was reported at 1,000 m (95.01 μM TE. g^–1^).

The PCA on Figure 7B displays the correlation between altitudes, antioxidants, and their activity. A total of 1,200 m altitude was characterized by ABTS activity, caffeine, and total phenolics due to the high concentrations detected at that altitude. A similar characterization was seen for 1,000 m, however, with DPPH activity. Total phenolics were grouped with caffeine and chlorogenic acid, and trigonelline was grouped with ABTS activity.

## Discussion

The aim was to characterize the microbial community and compounds profiles associated with fermented natural coffee from different altitudes. Out of the detected fungal community, yeast species were the most abundant possibly to the region’s high relative moisture and temperatures, which were within our ranges. Furthermore, this region has rainy summers (November to January) and cold and dry winters (June to August). The average annual rainfall ranges from 1,000 to 1,500 mm, and the average annual temperature ranges from 19 to 22°C (Campanha et al., 2017). Other factors that probably influenced yeast occurrence were the temperature inside our mass that varied from 21.6 to 25°C and SIAF conditions, becoming beneficial for their growth.

The coffee’s microbiota in this work varied at different altitudes. Lower altitudes favored bacterial richness, meaning that altitude is a factor that affects this microbial group. The high *Gluconobacter* abundances found in this work were also reported in a fermented natural processed coffee from Ecuador (De Bruyn et al., 2017). Therefore, the high abundances depend on the processing instead of the altitude. Acetic acid bacteria (AAB) are known to be strictly aerobic and capable of oxidizing alcohols, aldehydes, and sugars into carboxylic acids (Gomes et al., 2018). However, there was no correlation between *Gluconobacter* and acetic acid production in our work, but possibly other AAB, such as *Acetobacter*, were responsible for the high acetic acid production. A food fermentation with similar microbial dynamics as in coffee is cocoa (Schwan et al., 2015). Ho et al. (2018) have confirmed AAB’s role in cocoa fermentation, which involves acetic acid production (primary acid involved in cocoa fermentation), pH increase, and volatiles production. A bacterial genus capable of producing acetic acid is *Weissella*, which might have aided with the acetic acid production in this study since the genus was present at all altitudes. However, *Weissella* belongs to the lactic acid bacteria (LAB) group and is heterofermentative (producing acetic acid and lactic acid) (Lorenzo et al., 2018). According to Martins et al. (2020), among the isolated LAB from the Caparaó region, *Weissella paramesenteroides* were more abundant in natural coffees. Moreover, this genus has been found in coffee fruits from Taiwan (Leong et al., 2014), a wet-processed coffee from Colombia (de Oliveira Junqueira et al., 2019), and an Ecuadorian natural processed coffee (De Bruyn et al., 2017).

The LAB *Leuconostoc* was detected at all altitudes and showed a strong positive correlation with lactic acid, suggesting that this genus may be responsible for its production. Prior works testing the *Leuconostoc* genus’ potential in coffee have shown that they are incapable of producing pectinolytic enzymes (Avallone et al., 2002). However, they produce lactic acid as a primary compound during fermentation (De Bruyne et al., 2007). In coffee, species of *Leuconostoc* have been isolated from Ethiopian coffee fermentation (De Bruyne et al., 2007) and were abundant in a coffee fermentation performed at 1,329 m in Ecuador (De Bruyn et al., 2017).

For the first time, we report a high abundance of the Proteobacteria *Methylobacterium* in a natural processed coffee fermentation at 1,400 m. Limited information on its function during fermentation is provided, yet they are known as plant growth-promoting bacteria (Ponnusamy et al., 2017). There is no current information about their correlation with citric acid or the contribution to coffee fermentation. Yet, there was a positive correlation between *Methylobacterium* and citric acid, and where the highest content of this acid was detected, this genus was most abundant, possibly to its overproduction during Krebs Cycle. However, further studies must be done to understand their relation.

Similarly, it is the first time *Sphingomonas* (most abundant in 1,200 m fermented coffee), *Roseomonas*, *Fructobacillus*, and *Nakamurella* are reported in natural coffee processed fermentations. Still, some have already been identified on a wet-processed coffee fermentation (De Carvalho Neto et al., 2018).

According to Martinez et al. (2019), bacteria are the primary acid producers in wet fermentation. Therefore, in this work, the bacterial community from dry-processed coffees was correlated with acids. Acetic acid significantly predominated coffees from the Caparaó region. Bressani et al. (2018) reported similar results in a different region: coffee was fermented with fewer hours and another variety Catuaí at an altitude of 750–800 m. Citric acid is expected to be significant because it is a primary compound produced by any microorganism and enhances fruity flavors. Overall, coffees from 1,200 and 1,400 m favor acetic, malic, and citric acid content (Figure 3). Therefore, altitude affects their concentration, and they are expected in higher concentrations because they positively contribute to the beverage acidity (Buffo and Cardelli-freire, 2004). No detection of butyric and propionic acid indicates that 48 h is a proper time for SIAF fermentation and guarantees non-production of off-flavors (Silva, 2015; Haile and Kang, 2019b). As observed in our results, 1,200 m favored tartaric acid production and was correlated with the bacteria genera only found at that altitude and not in the other altitudes, which means that they might be responsible for the tartaric acid production or they might have stimulated the other genera to produce. Until now, no reports have shown their capacity to produce tartaric acid. Detection of tartaric acid in coffee is positively favorable since it produces fruity flavors like wine (Dziezak, 2016).

The bacterial communities were also correlated with volatile compounds because they contribute to their production. In this work, *Weisella* was correlated with Acetoin, and both were abundant at 1,000 m. Also, Acetoin was only detected at that altitude, suggesting that this genus may induce its production or other microorganisms. The same behavior was observed for compounds Methyl salicylate, 2-Propanone, 1- hydroxy-, and Benzeneacetaldehyde in the other altitudes with their respective genera.

Filamentous fungi and yeasts also compose the microbial communities of coffee during fermentation. The fungal diversity varied depending on the altitude and diversity index. Genus *Cystofilobasidium* (yeast) was the most abundant in all altitudes during SIAF conditions. Among the genus, *Cystofilobasidium ferigula* occurrence was at all altitudes with different relative abundances. This species was formerly designated as *Cryptococcus ferigula* and has been previously isolated from leaves submerged in a stream from a natural park in Portugal (Sampaio et al., 2007). C*ystofilobasidium infirmominiatum* is naturally found in cold habitats (Hu et al., 2014), suggesting that its predominance is due to the region’s characteristics and capability to resist low temperatures harvesting.

37.7% fungi were not present at all coffee growing altitudes, from which 17.16% represented the species that were only identified in coffee from 800 m, and 25.31, 12.29, and 5.74% in coffees from 1,000, 1,200, and 1,400 m. Therefore, even if coffee belongs to the same region, the altitude’s influence on the niches was evident. Since most abundant fungi species were yeast, those who are culturable can be isolated, studied, and use as inoculants for future fermentations in the Caparaó region. For this purpose, yeasts in high abundance such as *Meyerozyma caribbica* and *Wickerhamomyces anomalus* can be further used. The capacity to produce polygalacturonase and pectin lyase enzymes from *Wickerhamomyces anomalus* has already been demonstrated (Haile and Kang, 2019a). *Saccharomyces cerevisiae* was dominant in other coffee-producing regions (Silva et al., 2000; Evangelista et al., 2014b; Bressani et al., 2018), but not in this work.

The species below 1% abundance were clustered together, meaning they were not as influential as higher abundance species. Consequently, the NMSD plots showed that high abundance microbiota influences acids and volatiles contents, which was also confirmed in the Pearson correlations. The same behavior was seen for tartaric acid but with low abundance species.

The relative abundance of most filamentous fungi was within the 1%, which was expected since their populations usually dominate after several drying days due to reduced water activity (Silva et al., 2008b).

As expected, the alcohol group in this work had the highest number of compounds and content, possibly due to the high yeast abundance. Yeast uses the nitrogen compounds from amino acids to produce a pool of volatile alcohols (Dzialo et al., 2017), including phenylethyl alcohol, one of the alcohols detected in all altitudes. Coffees processed via the natural method in Evangelista et al. (2014b) and Bressani et al. (2020) had alcohols as the leading group during fermentation, and most were related to fruity odors. Like Bressani et al. (2020), high contents of 1-hexanol, 2-heptanol, benzyl alcohol, and benzaldehyde were also detected here. These volatiles compounds are essential for tea aroma (Ho et al., 2015).

In coffee, either alcohols or esters are significant because they confer the most sensed odor descriptors. In this study, low altitudes and microbiota are strongly associated with volatile alcohols; these were also the altitudes with the highest bacterial and fungal richness and probably influenced the alcohol quantity. Simultaneously, high altitudes and their microbiota are strongly associated with high contents of aldehydes and esters.

Caffeine, chlorogenic acid, and trigonelline concentrations in our work were in the same range as those previously detected at 800 m in Bressani et al. (2018). Caffeine is crucial because it confers bitterness to the beverage (Sunarharum et al., 2014). As for chlorogenic acids, they are responsible for pigmentation, astringency, and the production of volatile phenols (Duarte et al., 2010; Sunarharum et al., 2014). Trigonelline is responsible for the overall sensory perception. Most importantly, they all exert antioxidant properties. After fermentation, the coffees from higher altitudes contained higher concentrations of caffeine. Total phenolics are mainly composed of tannins and partly chlorogenic compounds (Farah and Donangelo, 2006). With the obtained results, it was observed that the concentration of the chlorogenic acid was only a small part of total phenolics concentration, being supported when correlated (Figure 7).

Hence, the antioxidant activity depends on time, temperature, nature of the substance, and concentration of antioxidants or other compounds (Yashin et al., 2013). Concerning our fermented coffees, the altitude that contained the highest content of total phenolics (i.e., 1,200 m) was the altitude with the highest antioxidant activity when measured by ABTS.

## Conclusion

This work microbial and chemical characterization revealed a new perspective of why coffee from the Caparaó region is different from other Brazilian regions. The altitude and other region characteristics drive shifts in the microbiota profile and abundance, favoring yeast communities during fermentation. Moreover, altitude and high abundance of microbiota affect acetic and citric acid concentration and volatile compounds. 800 m coffee favors bacterial richness, and 1,000 m favors fungal richness during fermentation under SIAF conditions. Yeast that resists low temperatures dominates the Caparaó region coffee’s (mainly from genus *Cystofilobasidium*). Dominant microbiota from different altitudes and controlled conditions by SIAF fermentations are the main drivers of biochemical compounds. Coffee from lower altitudes has higher contents of volatile alcohols, while high altitudes have higher esters, aldehydes, and total phenolic contents. Besides, the AAB function in coffee is still unknown; future approaches implementing AAB as inoculants need to be studied.

## Data Availability Statement

The datasets presented in this study can be found in online repositories. The names of the repository/repositories and accession number(s) can be found below: NCBI SRA BioProject, accession no: PRJNA706460, https://www.ncbi.nlm.nih.gov/bioproject/PRJNA706460/.

## Author Contributions

RS contributed to the experimental design and revision and edition of the manuscript. JS contributed to the study design and data collection. VP supervised and contributed to the manuscript revision. SM collected and analyzed the data, performed the statistical analysis, and wrote the manuscript’s first draft. All authors contributed to the article and approved the submitted version.

## Conflict of Interest

The authors declare that the research was conducted in the absence of any commercial or financial relationships that could be construed as a potential conflict of interest.
